# Ghrelin-Induced Orexigenic Effect in Rats Depends on the Metabolic Status and Is Counteracted by Peripheral CB1 Receptor Antagonism

**DOI:** 10.1371/journal.pone.0060918

**Published:** 2013-04-02

**Authors:** Francisco Alen, Inmaculada Crespo, María Teresa Ramírez-López, Nadine Jagerovic, Pilar Goya, Fernando Rodríguez de Fonseca, Raquel Gómez de Heras, Laura Orio

**Affiliations:** 1 Departamento de Psicobiología, Facultad de Psicología, Universidad Complutense de Madrid, Spain; 2 Instituto de Química Médica, Centro Superior de Investigaciones Científicas, Madrid, Spain; 3 Hospital Carlos Haya, Fundación Pública Andaluza para la Investigación en Málaga en Biomedicina y Salud (FIMABIS), Málaga, Spain; 4 Instituto de Salud Carlos III, Centro de Investigación Biomédica en Red de la Fisiopatología de la Obesidad y Nutrición (CIBEROBN), Madrid, Spain; University of Cordoba, Spain

## Abstract

Ghrelin is an endogenous regulator of energy homeostasis synthesized by the stomach to stimulate appetite and positive energy balance. Similarly, the endocannabinoid system is part of our internal machinery controlling food intake and energy expenditure. Both peripheral and central mechanisms regulate CB1-mediated control of food intake and a functional relationship between hypothalamic ghrelin and cannabinoid CB1 receptor has been proposed. First of all, we investigated brain ghrelin actions on food intake in rats with different metabolic status (negative or equilibrate energy balance). Secondly, we tested a sub-anxiogenic ultra-low dose of the CB1 antagonist SR141716A (Rimonabant) and the peripheral-acting CB1 antagonist LH-21 on ghrelin orexigenic actions. We found that: 1) central administration of ghrelin promotes food intake in free feeding animals but not in 24 h food-deprived or chronically food-restricted animals; 2) an ultra-low dose of SR141716A (a subthreshold dose 75 folds lower than the EC_50_ for induction of anxiety) completely counteracts the orexigenic actions of central ghrelin in free feeding animals; 3) the peripheral-restricted CB1 antagonist LH-21 blocks ghrelin-induced hyperphagia in free feeding animals. Our study highlights the importance of the animaĺs metabolic status for the effectiveness of ghrelin in promoting feeding, and suggests that the peripheral endocannabinoid system may interact with ghrelińs signal in the control of food intake under equilibrate energy balance conditions.

## Introduction

Ghrelin is a potent anabolic molecule with orexigenic and adipogenic actions. This hormone is produced in the periphery, mainly by the stomach but also by lower parts of the gastrointestinal tract [1], [2], [3]. The peripherally-produced ghrelin reaches the central nervous system via the activation of the vagus nerve. Within the central nervous system, ghrelin activates specific hypothalamic areas connecting with the orexigens neuropeptide Y, orexin, and Agouti-related peptide [4], [5] and regulates growth hormone (GH) secretion and energy homeostasis [6]. Ghrelin receptor (growth hormone secretagogue receptor, GHS-R1a) is located in the hypothalamus, nodose ganglia and gastrointestinal tract and its activation reduces energy expenditure, stimulates food intake and promotes gluconeogenesis and adipose tissue deposition [2], [7], [8]. In addition, feeding status appears to determine plasma ghrelin levels in humans [1]. However, the orexigenic actions of ghrelin administration are mainly known in the context of an equilibrated energetic balance but little is known about the role of this hormone in different feeding status where the energy intake is not equal to the energy expenditure.

Similar to ghrelin, the endogenous cannabinoid system (ECS) plays a major role in feeding control, energy homeostasis and metabolism [9], [10], [11], [12]. Both peripheral and central mechanisms for the ECS-mediated control of feeding have been described. The ECS controls food intake by a complex network of interactions with a wide variety of peptides or hormones, including orexin A-hypocretin 1 [13], [14], [15], cholecystokinin [16], oxytocin [17], leptin [18], and the melanocortin [19], [20] and opioid [21], [22] systems. Additionally, a functional relationship between central ghrelin and CB1 receptors has also been described, suggesting that the interplay between both systems may take place in the hypothalamic paraventricular nucleus [23], [24]. However, whether ECS and ghrelin may interplay also at a peripheral level is unknown.

Blockade of the CB1 receptors emerged as a potential valuable tool for the treatment of diet-induced obesity since chronic pharmacological blockade of CB1 receptors decreases food intake and body weight and improves lipid metabolism not only in animal models of obesity but also in humans [25], [26]. According to this beneficial profile, the CB1 antagonist SR141716A (Rimonabant, Acomplia®) was translated into therapy for humans [27]. Unfortunately, blockade of CB1 receptors was found to decrease food intake but also to induce central side effects such as depression [28] and, as a consequence, the compound was finally withdrawn from the market due to important psychiatric side effects (http://www.ema.europa.eu/humandocs/Humans/EPAR/acomplia/acomplia.htm). However, a better scientific understanding of the ECS distribution, mode of action and interplay with other systems may led us to rescue the positive effects of CB1 antagonism on food intake and metabolism regulation avoiding the limiting side central effects [29]. It is important to stress that the potency of SR141716A for inducing satiety in animals is similar to that for inducing anxiety ([30]; and this paper). In this regard, there is an increasing interest for developing new CB1 antagonist acting mainly peripherally [29]. The CB1 receptor distribution in the periphery include several tissues involved in metabolic actions such as liver, gastrointestinal tract, pancreas, or adipose tissue [31], [32], [33], [34]. Previous investigations allowed the identification of peripheral mechanisms for the ECS in the regulation of feeding behavior [35]. Systemic administration of low doses of SR141716A enhances c-fos expression in brainstem areas receiving vagal inputs as indicative of peripheral nervous system activation [16]. This c-fos expression in brainsteam areas was attenuated after higher doses of SR141716A, indicating a dose-dependent peripheral versus central actions of SR141716A [16]. In the last years, peripheral-restricted CB1 antagonists have been developed and tested for feeding inhibition [36], [37].

Because peripheral CB1 receptors may control appetite while no inducing anxiety [36], we further investigated whether peripheral-acting CB1 receptor antagonists may modulate other signals controlling appetite such as ghrelin. In this study we aim to determine the role of central ghrelin on food intake in rats under different metabolic status and investigate *in vivo* the functional interaction between ghrelin and the ECS, with emphasis in the role of peripheral CB1 receptors modulating the orexigenic signal of ghrelin. Our results provide a new perspective in the interaction between ghrelin and CB1 receptors and suggest that therapies using new peripheral-restricted CB1 antagonist might be efficacious for the treatment of obesity when ghrelin-inducing food intake signal is overactivated.

## Materials and Methods

### Ethic statement

The protocols for animal care and use were approved by the appropriate Committee of the Ethics of Animal Experiments of the Complutense University. Furthermore, all experimental procedures were carried out according to the European Communities Council Directive of 22 July 2003 (2003/65/CE) and current Spanish regulations of animal research (Real Decreto 1201/2005, BOE 21–10–2005). All efforts were made in order to minimize the number and suffering of the animals used.

### Animals

Experimental subjects were a total of 171 adult male Wistar rats (Harlan Ibérica, Madrid, Spain) weighting approximately 250–270 g on the beginning of experiments and housed individually. The animals were acclimated to the facility and handled before any experiment. Rats were kept at constant room temperature (22±1°C) and relative humidity (52±2%) in a 12 h light/dark cycle (lights on at 7:00 am) and had free access to water. The majority of the experiments were started around 11 am. *Free-feeding* animals had free access to standard pellets of food; *fasted rats* were totally deprived for food during 24 h before the experiment; and *chronic food-restricted* rats were limited daily for food intake until body weights reached 80% of free-feeding values (20–25 days). When body weights reached the target value, the amount of food was adjusted daily to maintain a constant body weight. Standard pellets were presented to the rats and food consumption was evaluated at different time points after the beginning of food presentation. The nutritional composition of the regular diet for rats was 16.10% crude protein, 3.10% crude fat, 5.10% crude ash, 3.90% crude fibre, and a pre-mixture of vitamins and minerals, dicalcium phosphate and calcium carbonate. Body weight and water intake were also evaluated before and after food tests. The number of animals per group was 9 rats for each treatment in each condition.

### Surgery

Rats were anesthetized with equithesin (3 mg/kg) and unilaterally implanted with a stainless-steel guide cannula (22-G, Nessler, Spain) for intracerebroventricular (i.c.v.) injections. Cannula was aimed at the right or left brain lateral ventricle and stereotaxically implanted according to coordinates determined from the rat brain atlas of Paxinos and Watson (1998) [38] (−0.92 mm antero-posterior; ±1.4 mm lateral to bregma, and 2.8 mm below the skull surface). The injector extended 1 mm beyond the end of the guide cannula. Animals were allowed 9 days post-operative recovery prior to any experimental manipulation. Extensive details on the methods have been published elsewhere [35].

### Drugs and treatments

Ghrelin (Tocris, Madrid, Spain) was dissolved in saline (99%) and dimethylsulfoxide (DMSO) (1%) and was administrated i.c.v. in brain lateral ventricle (0.25, 0.5 or 1 µg in 5 µl during 30 s) following a between-subject design with repeated measures at different time points. To ensure complete dispersal of the treatment we kept the injector *in situ* for additional 30 s.

SR141716A (N-piperidino-5-(4-chlorophenyl)-1-(2,4-dichlorophenyl)-4-methylpyrazole-3-carboxamide or Rimonabant) was kindly provided by Sanofi Recherche (Montpellier, France) and LH-21 [5-(4-chlorophenyl)-1-(2,4-dichlorophenyl)-3-hexyl-1H-1,2,4-triazole] was synthesized in our laboratory as described before [39]. Both SR141716A and LH-21 were suspended in a vehicle containing Tween 80 (1%) and saline (99%) and administered by intraperitoneal injection in a volume of 1 ml/kg prior to the i.c.v. injection of ghrelin or vehicle.

Rats received three habituation i.c.v. injections and two intraperitoneal injections (saline) before any treatment.

### Statistical analysis

Statistical analyses were performed using SPSS statistical package version 11.0 to Windows or GraphPad Prism (version 5.0) software. A three-way analysis of variance (ANOVA) was performed with three variables: metabolic status (between subjects) x ghrelin dose (between subjects) x time (within subjects). The two-way ANOVAs were performed followed by a Bonferroni *post hoc* test. All data are presented as mean ± standard errors of the mean and P<0.05 was considered statistically significant.

## Results

### Effect of i.c.v. ghrelin administration on food intake in rats with different metabolic status

We tested whether treatment with ghrelin (0, 0.25, 0.5 or 1 µg i.c.v.) increased feeding in animals under different metabolic status: animals ad lib fed (free feeding animals), food-deprived for 24 hours (fasted), and chronic food-restricted rats (n = 9 per group) at different time points (60 min and 120 min) post treatment. A 3-way ANOVA (metabolic status x ghrelin dose x time) revealed no overall interaction among the three factors (F_(6,96)_ = 0.44, P = 0.85). However, the test of between-subject effects revealed an interaction between the metabolic status and the dose of ghrelin (F_(6,96)_ = 7,15, P = 0.0001), being ghrelin treatment effective in free feeding animals but not in fasted or chronically deprived animals. The test of within-subject contrast revealed no effect of time and ghrelin doses (F_(3,96)_ = 0.28, P = 0.84) but an overall interaction between time and metabolic status (F_(2,96)_ = 10,79, P = 0.0001), being the time after food presentation more influencing in those conditions where the animals were restricted for food. A comparison among vehicle-injected rats in each condition using two-way ANOVA revealed an interaction between metabolic status and time (F_(2,24)_ = 5.45, P = 0.001), and main effects of time (F_(1,24)_ = 21.23, P = 0.0001), and metabolic status (F_(2,24)_ = 191.4, P<0.0001). A Bonferroni *post hoc* test indicated significant differences among the control groups in each metabolic condition, consuming more food the chronically food-restricted rats, following by the fasted group and free feeding rats, respectively.

**Figure 1:**
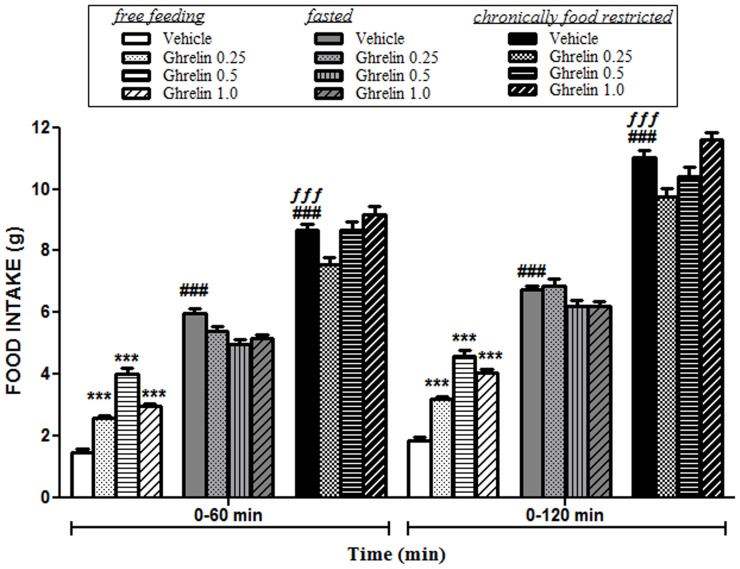
Effects of ghrelin (0.25 µg, 0.5 µg and 1.0 µg, i.c.v.) on accumulated food intake in free feeding, 24 h food-deprived (fasted) or chronically food-restricted animals 60 min and 120 min after administration. Free-feeding animals (n = 9 in each group) had free access to standard pellets of food; fasted rats (n = 9 in each group) were totally deprived for food during 24 h before the experiment; and chronic food-restricted rats (n = 9 in each group) were limited daily for food intake until body weights reached 80% of free-feeding values (20–25 days). The amount of ingested food in vehicle-injected animals was higher in the food-restricted group, following by the fasted group and free feeding rats, respectively. Ghrelin administration induced hyperphagia at any dose tested exclusively in animals fed ad libitum. No effect of ghrelin administration was observed in fasted or food-restricted animals. Data are means ± SEM of accumulated food intake. Different from free feeding vehicle-injected rats: ^###^P<0.001. Different from fasted vehicle-injected rats: ^£££^P<0.001. Different from vehicle-injected rats in the same condition (free feeding): *P<0.05. **P<0.01. ***P<0.001.

A detailed analysis of the free feeding condition using a two-way ANOVA revealed a main effect of ghrelin dose (F_(3,70)_ = 154.4, P<0.0001) and main effect of time (F_(1,70)_ = 52,9, P<0.0001). The interaction between factors (dose x time) was found in the limit of significance (F_(3,70)_ = 2.67, P = 0.05). *Post hoc* analysis showed significant differences on food intake after ghrelin treatment at doses of 0.25 µg (P<0.001), 0.5 µg (P<0.001) and 1.0 µg (P<0.001) 60 min and 120 min after food presentation (Fig. 1).

No changes in water intake were found in any group tested (data not shown).

### EC_50_ of a dose-response curve of SR141716A on food intake and anxiety

**Figure 2:**
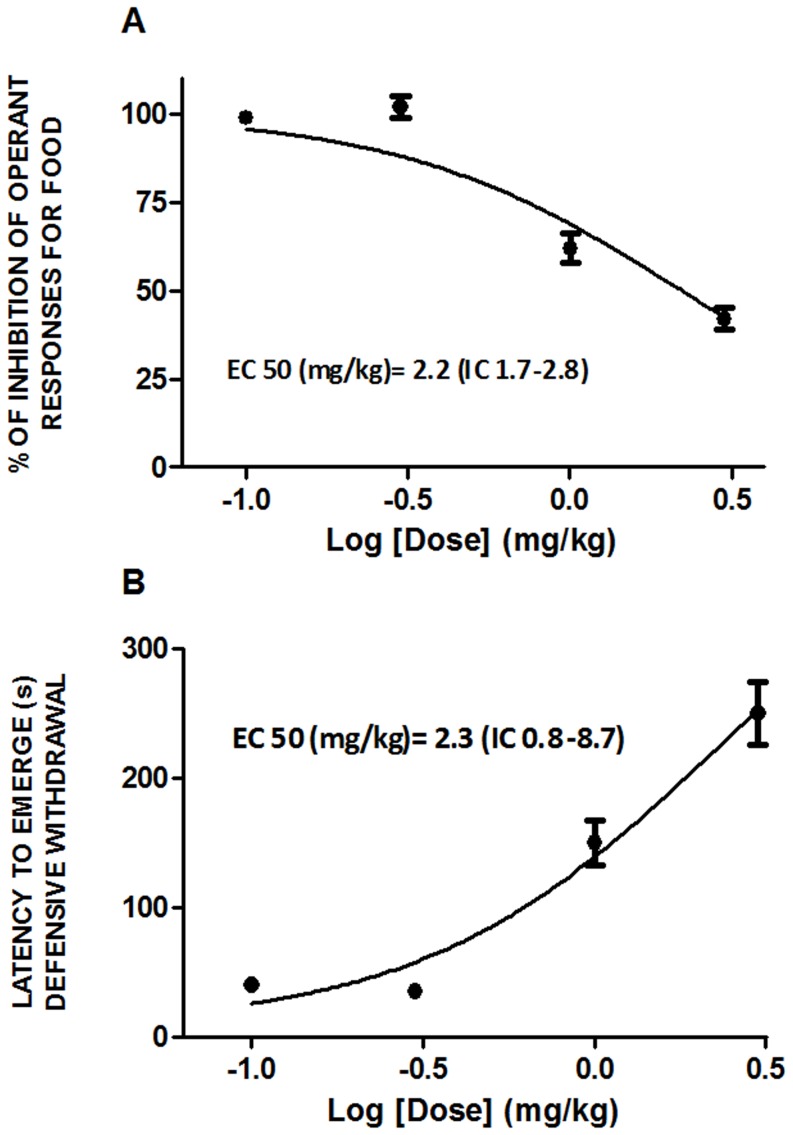
EC_50_ of a dose-response curve of SR141716A on food intake and anxiety. A) effect of SR141716A (0, 0.1, 1.0 and 3.0 mg/kg, i.p.) on responses for food; B) effect of SR141716A (0, 0.1, 1.0 and 3.0 mg/kg, i.p.) on anxiety, expressed as latency to emerge a defensive withdrawal behaviour. The figures represent a re-analysis of the data previously published [30]. The half maximal effective concentration (EC_50_) is 2.2 mg/kg and 2.3 mg/kg for food inhibition and induction of anxiety behavior, respectively.

We calculated the maximal effective concentration (EC_50_) of the CB1 antagonist SR141716A on food intake and anxiety (Fig. 2) by re-analysis of previous data published in [30]. The dose-response curves of SR141716A follow an inverted pattern for feeding and anxiety behaviors, being the EC_50_ for both conditions 2.2 mg/kg and 2.3 mg/kg, respectively. Taken these data into account, we choose an ultra-low SR141716A dose of 0.03 mg/kg (75 folds lower than the EC_50_) for our behavioral studies to test the effect of a subthreshold and subanorectic dose of this CB1 antagonist on ghrelin-induced hyperfagia. Therefore, this dose of SR141716A has nule effects on food inhibition and induction of anxiety (Fig. 2). Previous investigations provided evidence for a dose-dependent effect of SR141716A, being low doses effective for peripheral activation whereas highest doses clearly act at central level [16]. Consequently, the ultra-low dose of SR141716A chosen in this study allowed us to test the interaction between ghrelin and CB1 receptors at a peripheral level, as done elsewhere (Crespo I. et al., 2008).

### An ultra-low dose of SR141716A and the peripheral CB1 antagonist LH-21 both counteract ghrelin-induced orexigenic actions in free feeding animals

**Figure 3:**
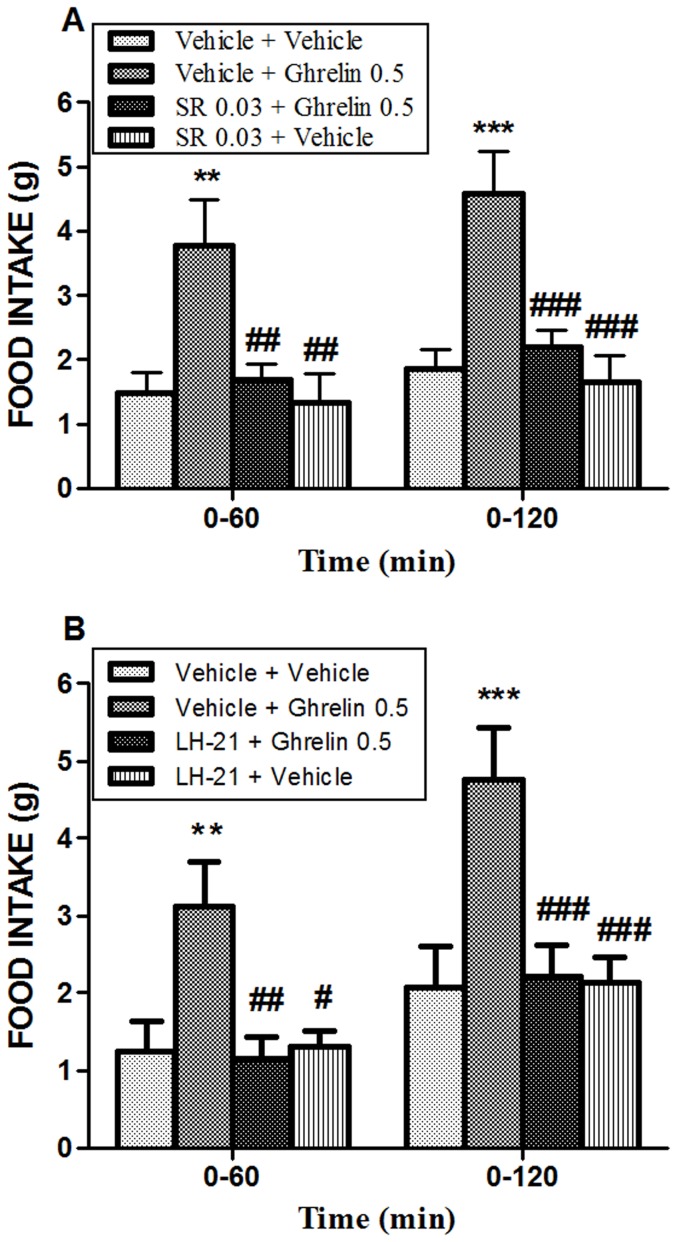
Pretreatment with an ultra-low dose of SR141716A or the peripheral CB1 antagonist LH-21 on the orexigenic effect induced by ghrelin in free feeding animals. Administration of ghrelin (0.5 µg, i.c.v.) increased food intake in free feeding animals. A) The subeffective dose of SR141716A (0.03 mg/kg, i.p) counteracted ghrelin-induced orexigenic effect. As expected, the ultra-low dose of SR141716A showed no effect on food intake in vehicle-treated animals. B) Pretreatment with LH-21 (3 mg/kg, i.p.) counteracted the increase in food intake induced by ghrelin and had no effect in vehicle-treated rats. Data are means ± SEM of 6–10 determinations per group. Different from vehicle-injected rats: *P<0.05. ***P<0.001; different from ghrelin treatment: ^#^ *P*<0.05, ^##^ *P*<0.01, ^###^ *P*<0.001.

Since ghrelin has orexigenic activity only in free feeding animals we choose this condition to test the effect of a subthreshold dose of SR141716A on ghrelin-induced hyperphagia (Fig. 3A). Free feeding animals were pre-treated with an ultra-low dose of SR141716A (0.03 mg/kg, i.p.) 10 min before the i.c.v. injection of the maximal ghrelin effective dose (0.5 µg) inducing feeding. SR141716A dose was selected based on its *Ki* for feeding and anxiety behaviors ([13]; and this paper). A two-way ANOVA revealed no interaction (within factors time and treatment F_(3,66)_ = 0.12), no effect of time (F_(1,66)_ = 2.64), but there was an overall effect of treatment (F_(3,66)_ = 14.90). The *post hoc* analysis showed that ghrelin increases feeding compared with vehicle at 60 min (*P*<0.01) and 120 min post-treatment (*P*<0.001). Pre-treatment with the non-anorectic dose of SR141716A counteracted the orexigenic effects of ghrelin 60 min (*P*<0.01) and 120 min (*P*<0.001) post- administration. As expected, the ultra-low dose of 0.03 mg/kg of SR141716A produced no significant differences on food intake versus vehicle-treated animals (*P*>0.05) (Fig. 3A). No change was found either on body weight or water intake in those animals (data not shown).

Additionally, free feeding animals were pretreated with systemic injections of the peripheral CB1 antagonist LH-21 (3.0 mg/kg, i.p.) 15 min before an i.c.v. injection of ghrelin (0.5 µg, i.c.v.) (Fig. 3B). A two-way ANOVA revealed no interaction (F_(3,37)_ = 0.36) but an overall effect of treatment (F_(3,37)_ = 12.26) and time (F_(1,37)_ = 11.43). The *post hoc* test indicate that ghrelin treatment increases feeding at 60 min (*P*<0.01) and 120 min post-treatment (*P*<0.001) compared with vehicle-injected animals. LH-21 pretreatment blocked the orexigenic effects of ghrelin in free feeding animals 60 min (*P*<0.01) and 120 min (*P*<0.001) after injection, and produced no effect on food intake in vehicle-treated animals (*P*>0.05, n.s.) (Fig. 3B).

## Discussion

The main findings of this study are: a) ghrelin-induced orexigenic effects depends on the metabolic status of the animal, being effective in free feeding conditions but having no effect in animals under negative energy balance conditions such as fasted or chronically food-restricted rats; b) blockade of CB1 receptors at extremely low doses (not centrally acting) or with peripheral-restricted CB1 antagonists is able to counteract the hyperphagic effect of central ghrelin administration in free feeding animals.

We observed that orexigenic effects of i.c.v. ghrelin administration are only manifested under equilibrate energy balance conditions (free feeding) being ghrelin ineffective in animals with negative energy balance (fasted or chronically food-restricted animals). The increase in food intake after central ghrelin administration occurs rapidly at the first 60 minutes, according to the physiological role of this peptide hormone in meal initiation in humans [40], [41]. Feeding status appears to determine plasma ghrelin levels in humans [1]. It is known that total ghrelin levels increase after fasting and are suppressed within minutes by refeeding or enteral nutrient administration [41], [42], [43]. Specifically, plasma ghrelin levels increase by 31% after 12 h fasting and they are reduced by 22% immediately after feeding [1]. Therefore, plasma ghrelin levels are upregulated under negative energy balance conditions including starvation, whereas they are down-regulated under conditions of positive energy balance [42], [43], [44]. In line with the aforementioned studies, the lack of ghrelin administration effect in feeding behaviour found in fasted or chronically food-restricted animals in this study might be explained as the result of a ceiling effect caused by high ghrelin levels during these negative energy balance's states. Indeed, plasma ghelin-like immunoreactivity levels were markedly elevated in patients with anorexia nervosa and negatively correlated with body mass indexes [1]. Alternatively, obese individuals showed decreased levels of total ghrelin as a possible adaptation to the continued negative energy balance ([43]; but see [45]). Interesting, energy restriction also modulates CB1 expression in vagal afferent neurons [46], suggesting that metabolic status may influence both ghrelin and endocannabinoid systems which may interact in the control of feeding behavior as discussed later. A detailed understanding of the principles of ghrelin action under different metabolic conditions and its relationship with other peptides systems such as the ECS could be invaluable to understand the physiological role of this hormone controlling appetite.

The role of the ECS in the regulation of food intake has been widely studied in the past few years. However, the relative contribution of central acting versus peripheral ECS in the modulation of food intake and energy balance is still focus of intense research (reviewed in [12], [29]). The SR141716A-mediated inhibition of feeding by blockade of CB1 receptors at central level directly correlates with its anxiogenic activity. In this study we show that the EC_50_ of SR141716A on food inhibition is comparable to its EC_50_ for induction of anxiety. This fact is actually a handicap for the use of CB1 antagonists in the treatment of obesity and related disorders. According to this, to potentiate the peripheral contribution of CB1 receptors in the control of feeding and energy homeostasis emerges as a valuable alternative to treat obesity, due to the devastating psychiatric side effects found by blocking central CB1 receptors [9], [12], [29]. In order to test the nature of ghrelin and ECS interaction and the contribution of peripheral CB1 receptors in ghrelin-induced feeding, we choose an ultra-low dose of SR141716A, which is 75 folds lower than the EC_50_ for inhibition of feeding and, more importantly, for induction of central effects such as anxiety (this paper, and [30]). In our study we showed that an intraperitoneal ultra low-dose of SR141716A is able to counteract the orexigenic effect of i.c.v. ghrelin administration in free feeding animals. This result suggests that there is a powerful cross-talk *in vivo* between ghrelin and ECS in the modulation of feeding and that this interaction may be occurring at a peripheral level, since low doses of SR141716A are more associated with activation of peripheral sensory terminals whereas higher doses might be related to central effects [16], [35]. A functional interaction between ghrelin and ECS has been previously described by others showing that SR141716A (1 mg/kg, i.p.) abolished the orexigenic effect of intra-hypothalamic injection of ghrelin [24]. However, such interaction has been understood as a consequence of a central cross-talk between ghrelin and ECS in hypothalamic areas [23], [24]. As presented in Fig. 2 of this paper, the dose of 1 mg/kg of SR141716A (abscisses 0) used in the mentioned paper is a centrally acting dose that reduce feeding effectively and induces anxiety behaviors. Our study shows the effectiveness of a SR141716A ultra-low dose (that neither reduce feeding by itself nor is anxiogenic) on ghrelin-induced hyperphagia.

Additionally, we tested LH-21, a triazol derivative with poor penetration into the central nervous system [37], in the hyperphagia induced by ghrelin in free feeding animals. We previously demostrated that LH-21 induces appetite reduction in fasted rats and in free feeding animals under standard diet (and even more efficiently under high fat diet) when treated chronically with this peripheral CB1 antagonist [37], [47]. Our results in the present study indicate that LH-21 induces no effects in feeding by acute administration in free feeding animals (vehicle-treated rats), but it is highly effective blocking ghrelin-induced hyperphagia in those animals. This latest result, together with the blockade of ghrelin actions by subanorectic doses of SR141716A, suggests that the interplay between ghrelin and ECS may involve peripheral CB1 receptors. Some evidences for a peripheral influence of CB1 receptors on ghrelin actions have also been documented, giving some insight into our results. First of all, blockade of peripheral CB1 receptors decreases central actions of ghrelin such as ghrelin-induced growth hormone secretion, by a CB1 receptor mediated inhibition of hypothalamic GHRH mRNA expression that requires intact vagal afferent fibers [48]. Secondly, SR141716A reduce circulating plasma ghrelin levels in fed rats suggesting a modulatory control of peripheral CB1 receptors on ghrelin secretion by the gastrointestinal tract [49]. Thirdly, CB1 receptors and GHS-R1a receptors are both expressed in the nodose ganglia [7], [46], [50] and, precisely, intact vagal afferents neurons appears to be required for the orexigenic effects of ghrelin [50], [51], [52] and the anorectic effect of SR141716A ([35], but also see [53]). Whether both CB1 and GHS-R1 receptors co-localize anywhere in the ascending pathways from the periphery to the hypothalamus is still unknown, although both ghrelin and ECS appears to modulate the intracellular AMP-activated protein kinase in peripheral tissues and in the hypothalamus [54], [55]. It is possible that CB1 receptors and GHS-R1a receptor co-localize en the vagus nerve (and even in the hypothalamus) to modulate the same intracellular pathway. In this case, ghrelin and endocannabinoids may show an orexigenic synergic effect due to a signal amplification of one to the other in a heterodimer-like manner. Blockade of CB1 receptors may alter this amplified signaling. Specifically, SR141716A is an inverse agonist at CB1 receptors that presumably activates the opposite intracellular cascade than the agonist, and thus, binding of SR141716A to CB1 receptors may interfere with the activating pathway of ghrelin for feeding. LH-21 is a neutral antagonist that may then act via allosteric modulation of the heterodimer. Up to date, CB1 receptors has been found to generate heterodimers with G-protein coupled receptors involved in motivational behavior and feeding, including with mu-opioid [56], dopamine D2L [57] and orexin-1 receptors [14]. The hypothesis of heterodimerization suggests that ghrelin may be a more potent agonist of the GHS-R1/CB1 heteromer compared with the activation of the homomer or the single ghrelin receptor, as it happens with orexin A, which shows a 100-fold higher agonistic activity at OX1/CB1 heteromers compared with the orexin 1-receptor homomer binding [15], [58] and, thus, administration of SR141716A causes a decrease in the potency of orexin A in cells co-expressing both CB1 and OX1 receptors [14]. Similarly, the GHS-R1a receptor dimerization with other G-protein-coupled receptors involved in appetite regulation and food reward has been described elsewhere [59]. Thus, it is feasible to think that direct/indirect interaction of both CB1 and GHS-R1a receptors through functional heterodimerization might contribute to the effects observed. However, this hypothesis has to be determined. Additionally, it is known that ghrelin prevents the CB1 receptor downregulation in the human nodose ganglia induced by refeeding [7], indicative of the complexity of the ECS-ghrelin interaction. Further studies are needed to ascertain the specific nature of such interaction. Altogether, our present results and the results exposed above indicate that the orexigenic effect of ghrelin is not a consequence of a simple interaction of this hormone with its receptors and other systems within hypothalamic areas, whereas it requires intact peripheral connections with other peptides systems, such as the ECS, presumably in vagal afferent neurons, in order to efficiently act on feeding behavior.

In our study we can not exclude that the ECS-ghrelin interaction takes place directly in hypothalamic areas. Within the hypothalamus, one of the main areas involved in energy balance regulation, CB1 receptors are expressed in the arcuate nuclei, the paraventricular nuclei and the lateral hypothalamic area [60], [61] and GHS-R1a receptors are expressed in paraventricular, dorsomedial and ventromedial nucleus and arcuato [8], [62]. Interestingly, hypothalamic areas containing CB1 and GSH-R1 receptors such as the arcuate nuclei are not completely protected by the blood brain barrier because of its crucial role for the regulation of energy homeostasis [63]. It is to note that hypothalamic CB1 receptors are expressed in a relative low quantity levels but its targeting shows a high efficiency functional correlate [64]. It is possible then that the CB1 receptor interaction with other peptide systems/hormons such as ghrelin is responsible of such functional efficiency in the hypothalamus. Nevertherless, although the openings in the blood brain barrier might suggest a hypothalamic interaction the lack of activity of LH-21 or SR151716A at the doses used in this study in the HHA axis or inducing anxiety [30], [36] weakens this hypothesis.

Taken together, our results show that ghrelin acts differently according to the metabolic status of the animal, inducing hyperphagia in free feeding animals but not in rats under negative metabolic conditions. Our study also suggests that the behavioral interaction between ghrelin and CB1 receptors in the control of food intake may occur at the peripheral level and, therefore, we propose that there is still a future for novel CB1 antagonists unable to cross the blood-brain barrier to counteract the orexigenic signal when ghrelin is overactivated.
